# Supplementary material for: Time-series transcriptome analysis identified differentially expressed genes in broiler chicken infected with mixed Eimeria species
Source: Front Genet. 2022 Aug 8;13:886781. doi: 10.3389/fgene.2022.886781 (PMC9393255; doi:10.3389/fgene.2022.886781)
Supplement: Supplementary file 2 [file DataSheet1.ZIP › 7dpi_GO.Gsea.1625106495923/GOBP_MITOCHONDRIAL_TRANSLATIONAL_TERMINATION.html]

Details for gene set GOBP\_MITOCHONDRIAL\_TRANSLATIONAL\_TERMINATION[GSEA]

|  || Dataset | TMM\_7dpi\_gct\_format\_7dpi\_gct\_format.Class\_4dpi.cls #PC\_versus\_NC.Class\_4dpi.cls #PC\_versus\_NC\_repos |
| Phenotype | Class\_4dpi.cls#PC\_versus\_NC\_repos |
| Upregulated in class | 1 |
| GeneSet | GOBP\_MITOCHONDRIAL\_TRANSLATIONAL\_TERMINATION |
| Enrichment Score (ES) | 0.6438547 |
| Normalized Enrichment Score (NES) | 3.0892155 |
| Nominal p-value | 0.0 |
| FDR q-value | 0.0 |
| FWER p-Value | 0.0 |
Table: GSEA Results Summary

  

Fig 1: Enrichment plot: GOBP\_MITOCHONDRIAL\_TRANSLATIONAL\_TERMINATION      
 Profile of the Running ES Score & Positions of GeneSet Members on the Rank Ordered List

  

| SYMBOL | TITLE | RANK IN GENE LIST | RANK METRIC SCORE | RUNNING ES | CORE ENRICHMENT || 1 | MRPS12 | na | 242 | 0.999 | 0.0168 | Yes |
| 2 | MRPS6 | na | 315 | 0.928 | 0.0451 | Yes |
| 3 | MRPL30 | na | 351 | 0.883 | 0.0750 | Yes |
| 4 | MRPL43 | na | 504 | 0.741 | 0.0897 | Yes |
| 5 | MRPL23 | na | 564 | 0.704 | 0.1109 | Yes |
| 6 | MRPL12 | na | 579 | 0.694 | 0.1354 | Yes |
| 7 | MRPL57 | na | 643 | 0.665 | 0.1548 | Yes |
| 8 | MRPL58 | na | 755 | 0.609 | 0.1681 | Yes |
| 9 | MRPL54 | na | 779 | 0.596 | 0.1883 | Yes |
| 10 | MRPS33 | na | 798 | 0.591 | 0.2088 | Yes |
| 11 | MRPL35 | na | 853 | 0.571 | 0.2254 | Yes |
| 12 | MRPL10 | na | 872 | 0.563 | 0.2448 | Yes |
| 13 | MRPL55 | na | 880 | 0.561 | 0.2650 | Yes |
| 14 | MRPS16 | na | 950 | 0.538 | 0.2792 | Yes |
| 15 | MRPL21 | na | 1141 | 0.472 | 0.2808 | Yes |
| 16 | MRPL15 | na | 1217 | 0.450 | 0.2912 | Yes |
| 17 | MRPS10 | na | 1264 | 0.437 | 0.3035 | Yes |
| 18 | MRPS28 | na | 1280 | 0.432 | 0.3183 | Yes |
| 19 | MRPL40 | na | 1282 | 0.431 | 0.3342 | Yes |
| 20 | MRPL45 | na | 1312 | 0.427 | 0.3476 | Yes |
| 21 | MRPS23 | na | 1344 | 0.420 | 0.3606 | Yes |
| 22 | MRPL46 | na | 1380 | 0.409 | 0.3729 | Yes |
| 23 | MRPL28 | na | 1382 | 0.409 | 0.3879 | Yes |
| 24 | MRPL9 | na | 1437 | 0.396 | 0.3981 | Yes |
| 25 | MRPL3 | na | 1475 | 0.387 | 0.4093 | Yes |
| 26 | MRPL42 | na | 1521 | 0.375 | 0.4195 | Yes |
| 27 | MRPL19 | na | 1535 | 0.370 | 0.4321 | Yes |
| 28 | MRPL47 | na | 1550 | 0.367 | 0.4445 | Yes |
| 29 | MRPL1 | na | 1639 | 0.348 | 0.4501 | Yes |
| 30 | MRPS17 | na | 1651 | 0.345 | 0.4619 | Yes |
| 31 | MRPL34 | na | 1672 | 0.339 | 0.4728 | Yes |
| 32 | MRPS35 | na | 1686 | 0.336 | 0.4842 | Yes |
| 33 | MRPS11 | na | 1692 | 0.335 | 0.4962 | Yes |
| 34 | MRPL33 | na | 1696 | 0.333 | 0.5083 | Yes |
| 35 | MRPS18C | na | 1729 | 0.325 | 0.5177 | Yes |
| 36 | MRPL37 | na | 1736 | 0.324 | 0.5292 | Yes |
| 37 | AURKAIP1 | na | 1742 | 0.322 | 0.5407 | Yes |
| 38 | CHCHD1 | na | 1745 | 0.321 | 0.5525 | Yes |
| 39 | MRPL44 | na | 1799 | 0.307 | 0.5594 | Yes |
| 40 | MRPL2 | na | 1843 | 0.299 | 0.5669 | Yes |
| 41 | MRPS21 | na | 1864 | 0.296 | 0.5762 | Yes |
| 42 | MRPL16 | na | 1884 | 0.291 | 0.5854 | Yes |
| 43 | MRPL53 | na | 1984 | 0.273 | 0.5872 | Yes |
| 44 | MRPL41 | na | 2082 | 0.257 | 0.5886 | Yes |
| 45 | MRPL24 | na | 2093 | 0.254 | 0.5972 | Yes |
| 46 | MRPL13 | na | 2109 | 0.251 | 0.6053 | Yes |
| 47 | MRPS9 | na | 2193 | 0.238 | 0.6071 | Yes |
| 48 | MRPL48 | na | 2228 | 0.232 | 0.6129 | Yes |
| 49 | MRPL14 | na | 2284 | 0.224 | 0.6166 | Yes |
| 50 | MRPS14 | na | 2305 | 0.222 | 0.6231 | Yes |
| 51 | MRPS18A | na | 2363 | 0.213 | 0.6263 | Yes |
| 52 | MTRF1 | na | 2398 | 0.208 | 0.6311 | Yes |
| 53 | MRPS34 | na | 2496 | 0.193 | 0.6301 | Yes |
| 54 | MRPL18 | na | 2512 | 0.189 | 0.6359 | Yes |
| 55 | MRPS5 | na | 2649 | 0.170 | 0.6308 | Yes |
| 56 | MRPS25 | na | 2661 | 0.169 | 0.6362 | Yes |
| 57 | MRPL39 | na | 2682 | 0.166 | 0.6406 | Yes |
| 58 | MRPL22 | na | 2747 | 0.158 | 0.6411 | Yes |
| 59 | MRPL20 | na | 2783 | 0.153 | 0.6439 | Yes |
| 60 | MRPL38 | na | 2918 | 0.137 | 0.6377 | No |
| 61 | DAP3 | na | 3039 | 0.121 | 0.6321 | No |
| 62 | MRPL27 | na | 3152 | 0.107 | 0.6267 | No |
| 63 | MRPS7 | na | 3210 | 0.100 | 0.6256 | No |
| 64 | MRPL51 | na | 3225 | 0.098 | 0.6281 | No |
| 65 | MRPS22 | na | 3240 | 0.095 | 0.6305 | No |
| 66 | MRPS31 | na | 3424 | 0.071 | 0.6178 | No |
| 67 | MRPS26 | na | 3453 | 0.068 | 0.6179 | No |
| 68 | MRPL32 | na | 3822 | 0.025 | 0.5880 | No |
| 69 | MRPS27 | na | 3855 | 0.021 | 0.5861 | No |
| 70 | MRPS2 | na | 4677 | -0.068 | 0.5198 | No |
| 71 | ERAL1 | na | 4988 | -0.102 | 0.4977 | No |
| 72 | MRPS36 | na | 5609 | -0.160 | 0.4516 | No |
| 73 | MRPL17 | na | 5768 | -0.177 | 0.4449 | No |
| 74 | PTCD3 | na | 6171 | -0.221 | 0.4195 | No |
| 75 | MRPL50 | na | 6658 | -0.271 | 0.3888 | No |
| 76 | MTRF1L | na | 7465 | -0.357 | 0.3345 | No |
| 77 | MRPS30 | na | 8183 | -0.444 | 0.2908 | No |
| 78 | GFM2 | na | 10463 | -0.803 | 0.1296 | No |
Table: GSEA details [plain text format]

  

Fig 2: GOBP\_MITOCHONDRIAL\_TRANSLATIONAL\_TERMINATION      
 Blue-Pink O' Gram in the Space of the Analyzed GeneSet

  

Fig 3: GOBP\_MITOCHONDRIAL\_TRANSLATIONAL\_TERMINATION: Random ES distribution      
 Gene set null distribution of ES for **GOBP\_MITOCHONDRIAL\_TRANSLATIONAL\_TERMINATION**
